# Migration and tuberculosis transmission in Hamburg, Germany: insights from 25 years of molecular epidemiology

**DOI:** 10.1186/s13073-026-01750-7

**Published:** 2026-09-17

**Authors:** Nicole Ullrich, Roland Diel, Karen Meywald-Walter, Christian Schwarzbach, Matthias I. Gröschel, Martin Kuhns, Inna Friesen, Stefan Niemann, Viola Dreyer

**Affiliations:** 1 Research Center Borstel, Leibniz Lung Center, Molecular and Experimental Mycobacteriology, Borstel, Germany; 2 Institute for Epidemiology, University Medical Hospital Schleswig-Holstein, Kiel, Germany; 3 LungClinic Grosshansdorf, Airway Research Center North (ARCN), German Center for Lung Research (DZL), Großhansdorf, Germany; 4 Public Health Department Hamburg-Mitte, Hamburg, Germany; 5 Department of Infectious Diseases and Respiratory Medicine, Charité- Universitätsmedizin Berlin, Berlin, Germany; 6 German Center for Infection Research (DZIF), Partner Site Hamburg-Lübeck- Borstel-Riems, Borstel, Germany; 7 Research Center Borstel, Leibniz Lung Center, National and Supranational Reference Center for Mycobacteria, Borstel, Germany; 8 Phylogenomics, Phylodynamics and Host-Pathogen Interactions team, EPHE, PSL University, Paris, France; 9 Institut de Systématique, Evolution, Biodiversité, ISYEB, Muséum national d’Histoire naturelle, CNRS, Sorbonne Université, EPHE, Université des Antilles, Paris, France

**Keywords:** Migration, Tuberculosis, Host-adaptation

## Abstract

**Background:**

Tuberculosis (TB) epidemiology in low-incidence settings is increasingly influenced by the importation of *Mycobacterium tuberculosis* complex (Mtbc) strains from high-incidence regions. Globally, more virulent “generalist” lineages such as Lineage 2 (L2) and Lineage 4 (L4) contrast with geographically restricted “specialist” lineages (L1, L3, L5–L9), reflecting differences in transmissibility and adaptation. The introduction of strains of diverse lineages can alter local transmission dynamics and drug susceptibility patterns, underscoring the need for high-resolution genomic surveillance.

**Methods:**

We performed whole genome sequencing (WGS) on 3,131 Mtbc strains collected from patients with TB in Hamburg, Germany, over 25 years (1997–2021). We analysed population structure and transmission dynamics in relation to patients’ self-reported geographical origins.

**Results:**

Strains from major Mtbc lineages L1–L6 and M. bovis were detected, with L4 strains being most prevalent (74.6%, n = 2,337). Lineage distribution shifted over time: L4 strains decreased from 83.5% in the first five years to 63.0% in the last five years, while L3 strains increased from 4.8% to 18.1%. These changes correlated with an increasing number of foreign-born patients rather than enhanced transmission of strains of specific lineages. L4 strains accounted for most clusters (81%, 218/269). In-depth analyses revealed heterogeneity in transmission potential among L4 sublineages, underscoring the importance of sublineage-level resolution. Strains of sublineages L4.1.2.1, L4.8, and L4.3 were detected in patients born in over 15 regions each, with >57% of cases from Europe, supporting their representation as globally successful generalist lineages.

**Conclusions:**

Migration substantially increased the genetic diversity of the Mtbc population in Hamburg but did not fundamentally alter local transmission dynamics. Transmission remained lineage-specific and was predominantly driven by established L4 strains, suggesting that successful transmission is mediated by locally adapted sublineages. Predominant L4.1.2.1 and L4.8 sublineages occurred in individuals from a wide range of countries, supporting the generalist–specialist hypothesis at the sublineage level. These findings highlight the interplay between pathogen characteristics and host demographics in shaping transmission and the value of integrating genomic, demographic, and epidemiological data to distinguish imported cases from sustained local transmission in low-incidence settings.

**Supplementary Information:**

The online version contains supplementary material available at https://doi.org/10.1186/s13073-026-01750-7.

## Background

Tuberculosis (TB) remains a leading global health challenge, and is the deadliest disease caused by a single pathogen [1]. Its burden varies geographically, with high-burden areas such as South-East Asia (45% of all cases), Africa (24%) and the Western Pacific (17%) accounting for most TB cases in 2023 [1]. Germany is a low-incidence country, with 5.3 cases per 100,000 inhabitants, annually [2]. However, increased human mobility, including migration and international travel, is potentially a key driver of the cross-border introduction of pathogenic *Mycobacterium tuberculosis* complex (Mtbc) strains from high-incidence areas [1–4]. This influx of those “foreign” strains can complicate control efforts by introducing Mtbc strains from genetically diverse lineages with varying transmissibility, virulence, and drug susceptibility profiles [5]. Understanding how imported strains influence local transmission dynamics is therefore critical for tailoring effective TB surveillance, prevention, and control strategies in low-incidence settings. Indeed, the Mtbc includes both human-adapted pathogen species, such as *Mycobacterium tuberculosis* (Mtb), and animal-adapted pathogen species (e.g. *M. bovis*) [6–9]. So far, ten human-adapted lineages (L) have been defined [10–14], with some being geographically widespread (“generalists”, such as L2 and L4) and others being regionally restricted (“specialists”, such as L1, L3, L5, and L6) [15–17]. Strains of L1 and L3 occur mainly around the Indian Ocean, whereas strains of L5 and L6 are mainly isolated from West Africa [11, 18].

Strains of geographically widespread lineages are thought to be more virulent and transmissible across diverse populations, whereas strains of geographically restricted lineages may be adapted to specific host populations and less transmissible elsewhere [15–17]. However, at the sublineage level, not all L4 strains are geographically widespread; strains of some sublineages are confined to certain countries and are rarely isolated elsewhere [17]. Stable host-pathogen associations among secondary active TB cases have been observed previously in large cities such as San Francisco or Montreal [15, 19]. Previous studies suggest that specialist lineages transmit more effectively within their sympatric populations, whereas strains of generalist lineages can cause outbreaks in diverse host settings, potentially altering local population structures [16, 18–21]. However, longitudinal data on the evolution of these dynamics in low-incidence areas are limited.

To address this gap, we analysed the population structure and transmission patterns in Hamburg over 25 years using whole-genome sequencing (WGS) of 3,131 Mtbc strains from TB patients. Our study investigates how host self-reported ancestry influences transmission and examines the distribution of strains of L4 sublineages to understand their geographical characteristics and potential impact on TB epidemiology in a low-incidence setting.

## Methods

### Study population

We have enrolled all culture-confirmed TB cases reported to the Hamburg Public Health Department Holstein between 1997 and 2021. This data belongs to a prospective population-based molecular epidemiological surveillance that has been conducted in Hamburg since January 1, 1997. The molecular epidemiological studies were embedded in mandatory routine surveillance and contact investigation work performed by the public health offices according to the legal mandate of the German infectious diseases law and approved by the Hamburg commissioner for data protection. Routinely, isolated strains derived from patient material were sent to the National Reference Laboratory for Mycobacteriology in Borstel, Germany. WGS of all culture-confirmed strains (described below) resulted in 3,131 samples which were included in further analysis. Case data were collected prospectively by staff of the local health authorities as part of routine TB case investigations, in accordance with legal reporting requirements, using a standardized questionnaire. The study is registered and approved by the Ethics Committee of the University of Lübeck (AZ-19-071) and was conducted in accordance with the Declaration of Helsinki.

### DNA extraction

After culture confirmation, mycobacterial DNA was extracted using the cetyltrimethylammonium bromide (CTAB)-lysozyme method as described previously [22].

### Whole genome sequencing

Extracted genomic DNA of the Mtbc strains were processed using the Nextera XT library preparation kit, following the manufactures instructions and sequenced with one of Illumina’s (San Diego, CA, USA) sequencers MiSeq, HiSeq or NextSeq 500. Strains were sequenced paired-end with a minimum average genome coverage of 50x. Raw read data were processed with MTBSeq v.1.1.0 using standard parameters [23]. Phylogenetic lineages (Mtbc lineages and known Beijing subgroups) were inferred from specific single nucleotide polymorphisms (SNPs) based on Coll et al. [10]. and Merker et al. [24]. Cluster analysis was performed using the core genome multi-locus sequence typing (cgMLST) method using MBioSEQ Ridom Typer software (Bruker), as described previously [25]. A minimum spanning tree was calculated by ignoring the pairwise missing values. To cover recent transmissions (referring to Meehan et al. [26]), we have used a cluster alert of five allele differences (d5). To compare our genomic sequences with datasets from other studies/regions, we have used the TB subdivision of the EnteroBase application [27] and searched for similar Sequence Types (STs; ≤ 12 distances) of our major clustered strains.

### Phylogenetic analysis of L4

For the L4 population structure, we calculated a maximum-likelihood (ML) tree using a concatenated sequence alignment (PE/PPE/PGRS genes and drug-resistance-associated genes were excluded) that we produced with the MTBseq “TBjoin” command and the IQtree software [28]. The automated ModelFinder option and ascertainment bias correction was used. We employed ultrafast bootstrap approximation with 1,000 replicates combined with a further optimizing step to reduce the risk of overestimating the branch support. The phylogenetic tree was mid-point rooted using FigTree v1.4.4 [29] and annotated using the online tool EvolView [30].

### Statistics

For all statistical analyses we have used the statistics software R (version 3.6.1 and 4.2.0) [31]. Descriptive statistics were performed for patient demographics, as well as for lineages, resistance categories, and clustering status of Mtbc strains. We used Chi-squared, Mann-Kendall Trend and the Wilcoxon-signed rank test, as well as the Pearson correlation coefficient. P-values < 0.05 were taken as significant characteristics. We grouped countries of birth into regions according to the United Nations statistics division (https://unstats.un.org/unsd/methodology/m49*).* In order to avoid small case numbers and facilitate the analysis, sublineages of L4 were defined as described above according to Coll et al. [10], subdivisions of L4.3 were all summarized as L4.3; L4.6.1.1 and L4.6.1.2 were summarized as L4.6.1; L4.6.2 and L4.6.2.1 were summarized as L4.6.2; L4.1.1, L4.1.1.1, L4.1.1.2 and L4.1.1.3 were summarized as L4.1.1.

## Results

**Fig. 1:**
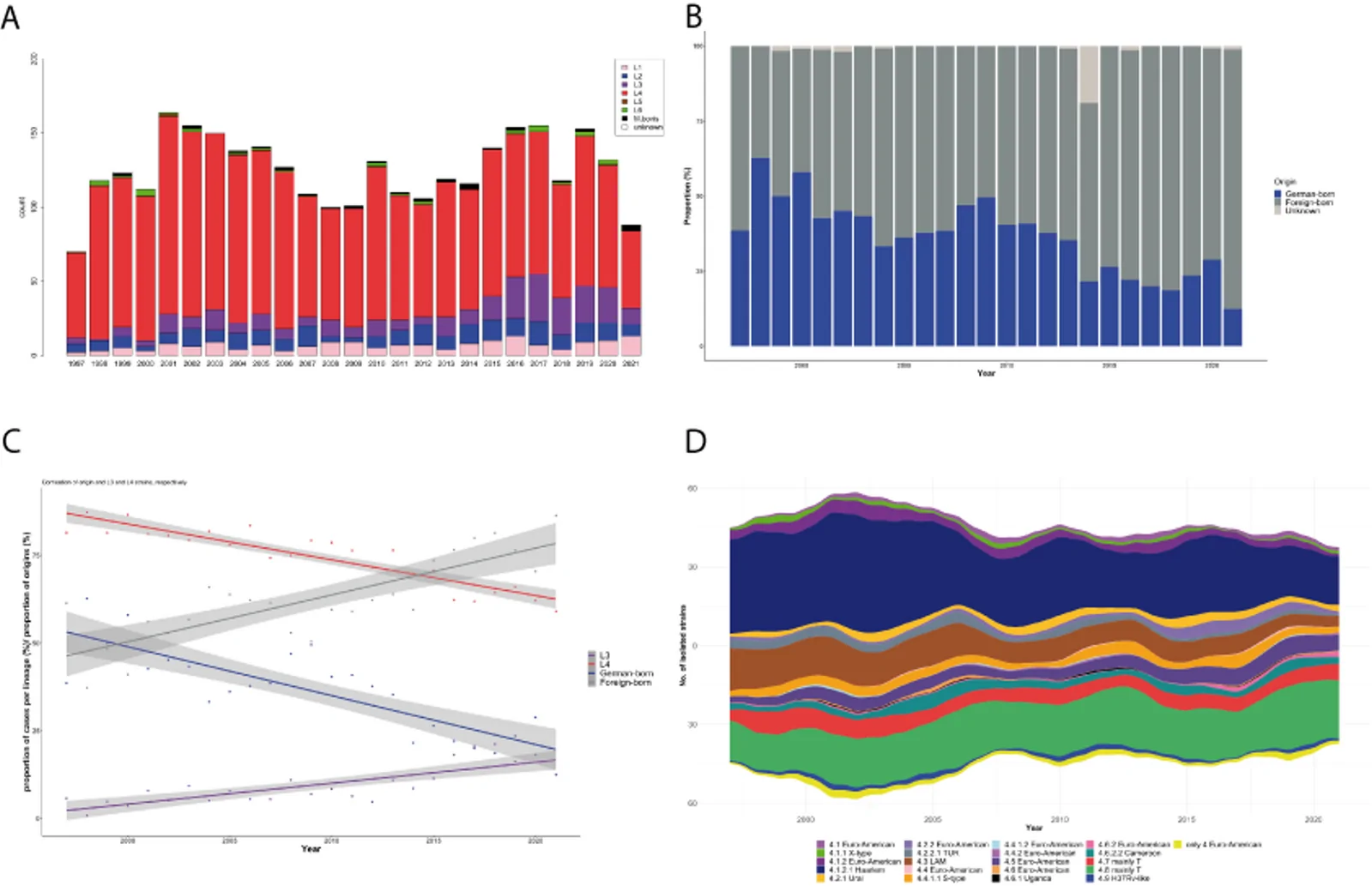
**A**: *Mycobacterium tuberculosis* complex (Mtbc) lineage distribution per study year, 1997–2021, *n* = 3,131. Strains infecting people in Hamburg, Germany, displayed in case numbers per lineage per year. Lineage 1 is displayed in pink, Lineage 2 in blue, Lineage 3 in purple, Lineage 4 in red, Lineage 5 in brown, Lineage 6 in green and *Mycobacterium bovis* in black. **B**: Proportion of German- and foreign-born tuberculosis (TB) patients in Hamburg, Germany, per year from 1997 to 2021. Proportions of TB cases of German-born patients are indicated in blue; proportions of foreign-born patients are indicated in grey. Unknown origin of the patients is indicated in beige. **C**: Correlation of patients self-reported ancestry in Hamburg, Germany, with Mtbc Lineage 3 strains and Lineage 4 strains, respectively, 1997–2021. Whereas proportions of Lineage 3 strains (purple) increase over time, in accordance to the proportion of foreign-born patients (grey), proportions of Lineage 4 strains (red) decrease over time in accordance to the proportion of German-born patients (blue). **D**: Streamgraph of Mtbc Lineage 4 sublineages in Hamburg, Germany, 1997–2021, *n* = 2,337. Number of strains of all Lineage 4 sublineages are displayed per year. Sublineages were categorised based on Coll et al. [10] as described in the method section

Between 1997 and 2021, we analysed 3,131 Mtbc strains isolated from culture-confirmed TB patients notified to the local Public Health authority in Hamburg, Germany. Annual numbers varied over the years between 70 in 1997 and 164 in 2001 (Median 124, interquartile range [IQR] 31; Fig. 1A) with a female: male ratio of 1:1.7 (Table 1). During the study period, the proportion of foreign-born patients increased significantly from 37% in 1998 to 86% in 2021 (Mann-Kendall trend test, *p* < 0.001), reflecting substantial demographic changes within the affected population (Fig. 1B).

**Table 1:** Study population in Hamburg, Germany, and basic *Mycobacterium tuberculosis* complex strain characteristics, 1997–2021, *n* = 3,131

Characteristic	Count	Percentage (%)
**Sex**		
Male Female undefined	1,967 1,161 3	63 37 0
**Origin**		
Foreign-born German-born unknown	1,964 1,131 36	63 36 1
**Age**		
< 10	50	1.6
11–20	192	6.1
21–30	629	20.1
31–40	610	19.5
41–50	490	15.6
51–60	409	13.1
> 60	737	23.5
unknown	14	0.4
**Resistance**		
Susceptible	2,634	84.1
non MDR	398	12.7
RR	17	0.5
MDR	64	2.0
Pre-XDR	15	0.5
XDR	3	0.1
**Lineage**		
L1	171	5.5
L2	240	7.6
L3	306	9.8
L4	2,337	74.6
L5	12	0.4
L6	32	1.0
*M. bovis*	32	1.0
unknown	1	0.1
**Clustering d5**		
Non-clustered	2,069	66
Clustered	1,062	34

Abbreviations: RR – Rifampicin resistant; MDR – Multi-drug resistant; XDR – Extensively drug resistant; L – Lineage; d5 – Allele distance 5

Concurrently, the composition of the population of the Mtbc strains changed (Fig. 1A). In general, patients were infected with strains of the major Mtbc Lineages 1 to 6 and *M. bovis* (Table 1). While L4 strains remained the dominant lineage throughout the study period (*n* = 2,337, 74.6%), their proportion decreased from 83.5% during the first five years to 63.0% during the last five years (Mann-Kendall trend test, *p* < 0.001). In contrast, the proportion of L3 strains increased from 4.8% to 18.1% (*p* < 0.001; Fig. 1C, Additional file 1: Table S1). Strains of other lineages showed lower variations in annual frequencies (Fig. 1A and Additional file 1: Table S1).

**Fig. 2:**
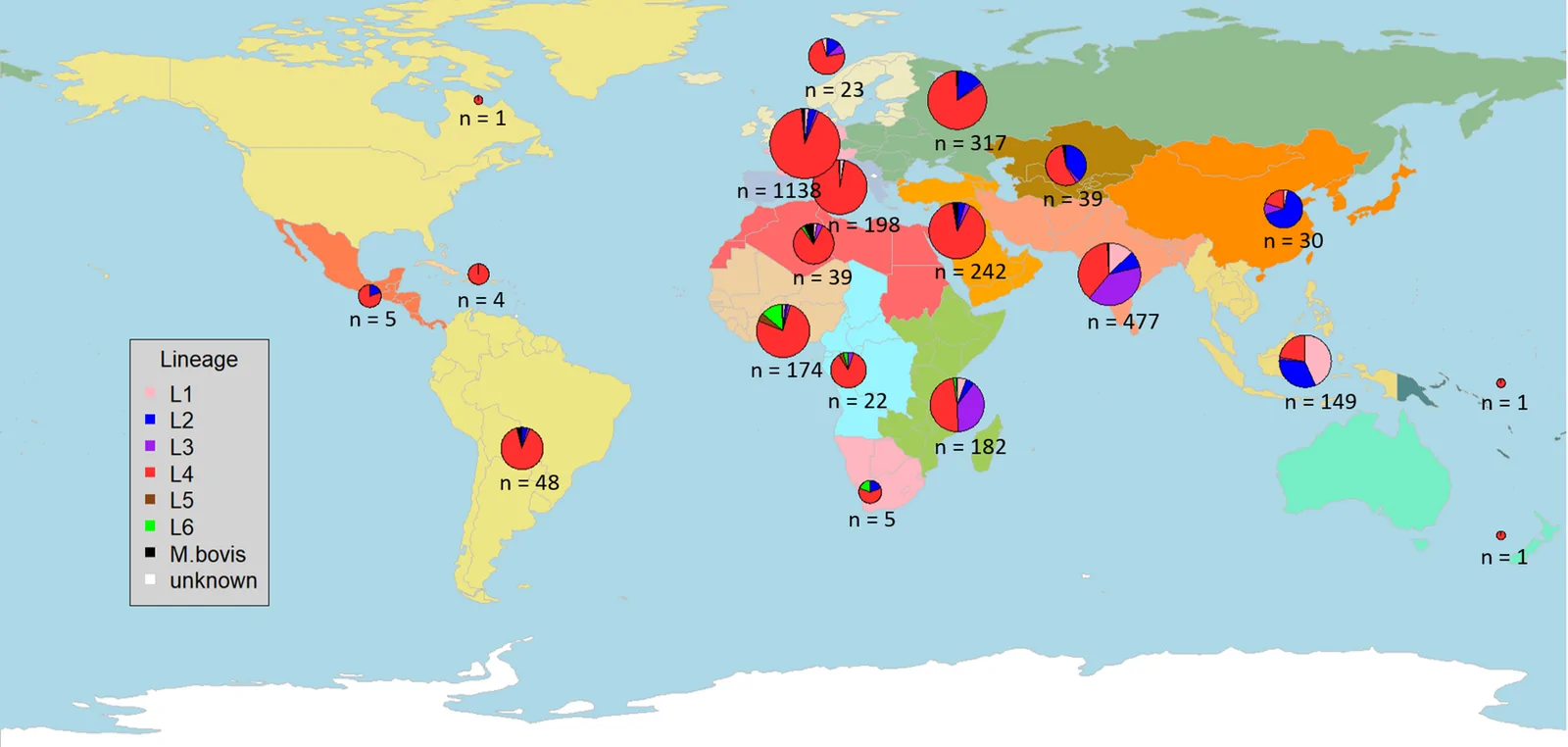
Lineage distribution of the *Mycobacterium tuberculosis* complex (Mtbc) strains at the patients’ self-reported ancestry grouped according to United Nations statistics division (https://unstats.un.org/unsd/methodology/m49). Each pie chart represents the population structure of Mtbc strains isolated from patients that had their self-reported ancestry in the respective region. Lineage 1 (L1) is displayed in pink, Lineage 2 (L2) in blue, Lineage 3 (L3) in purple, Lineage 4 (L4) in red, Lineage 5 (L5) in brown, Lineage 6 (L6) in green and *M. bovis* in black. Case numbers of each region are indicated below each pie chart

To determine whether these shifts in the population structure reflected local transmission or demographic changes, we analyzed the relationship between lineage distribution and patients’ self-reported ancestry based on their place of birth (Fig. 2). While 63% of all patients were foreign-born, this proportion was lowest among those infected with L4 strains (54%) and highest among those infected with L5 strains (100%, Table 2). The increasing proportion of L3 strains was strongly associated with the increasing proportion of foreign-born patients (*R* = 0.86, *p* < 0.001), whereas the declining proportion of L4 strains was strongly associated with the decreasing proportion of German-born patients (*R* = 0.75, *p* < 0.001, Fig. 1C). Further, we wanted to reach a higher resolution of the migration-associated influx of strains and their transmission. In order to do so, we grouped the countries of birth of all patients according to the United Nations statistics division. Following this, most patients reported their place of birth in Western Europe (36%), followed by Southern Asia (15.2%), Eastern Europe (10.1%), Western Asia (7.7%), Southern Europe (6.3%), and Eastern Africa (5.8%, Additional file 1: Table S2).

**Table 2:** Patient characteristics per strain type (Lineage) in Hamburg, Germany, 1997–2021, *n* = 3,131

	All	Lineage 1	Lineage 2	Lineage 3	Lineage 4	Lineage 5	Lineage 6	*M. bovis*
**Count**	3,131	171	240	306	2,337	12	32	32
**Origin**								
German-born	1,131 (36%)	22 (13%)	37 (15%)	15 (5%)	1041 (45%)	0 (0%)	2 (6%)	13 (41%)
Foreign-born	1,964 (63%)	146 (85%)	200 (83%)	288 (94%)	1,269 (54%)	12 (100%)	30 (94%)	19 (59%)
Unknown	36 (1%)	3 (1%)	3 (1%)	3 (1%)	27 (1%)	0 (0%)	0 (0%)	0 (0%)
**Average age** **[95% CI]**								
All	42 [41, 43]	35 [31, 39]	34 [33, 38]	34 [31, 37]	45 [43, 46]	30 [21, 38]	37 [29, 45]	55 [39, 71]
German-born	51 [50, 53]	50 [26, 60]	39 [31, 49]	34 [20, 61]	52 [50, 54]	-	31	72 [54, 74]
Foreign-born	37 [36, 39]	35 [31, 38]	33 [33, 37]	34 [31, 37]	39 [38, 40]	-	37 [31, 45]	42 [25, 55]
**Clustering d5**								
Clustered	1,062 (34%)	13 (8%)	62 (26%)	68 (22%)	914 (39%)	0 (0%)	5 (17%)	0 (0%)
Non-clustered	2,069 (66%)	158 (92%)	178 (74%)	238 (78%)	1,423 (61%)	12 (100%)	27 (84%)	32 (100%)
**Number of clusters**	269	6	21	22	218	0	2	0
**Medium cluster size [min – max]**	3.9 [2–32]	2.2 [2–3]	3.1 [2–8]	3.1 [2–19]	4.2 [2–32]	-	2.5 [2–3]	-
**Resistance**								
Sensitive	2,634 (84%)	145 (85%)	144 (60%)	258 (84%)	2,087 (89%)	0 (0%)	0 (0%)	0 (0%)
RR	17 (1%)	0 (0%)	1 (0%)	2 (1%)	9 (0%)	0 (0%)	0 (0%)	5 (16%)
Non MDR	398 (13%)	25 (15%)	45 (19%)	41 (13%)	216 (9%)	11 (92%)	32 (100%)	27 (84%)
MDR	63 (2%)	1 (1%)	36 (15%)	5 (2%)	21 (1%)	1 (8%)	0 (0%)	0 (0%)
Pre-XDR	15 (1%)	0 (0%)	12 (5%)	0 (0%)	3 (0%)	0 (0%)	0 (0%)	0 (0%)
XDR	3 (0%)	0 (0%)	2 (1%)	0 (0%)	1 (0%)	0 (0%)	0 (0%)	0 (0%)

Abbreviations: RR – Rifampicin resistant; MDR – Multi-drug resistant; XDR – Extensively drug resistant; CI – Confidence interval; d5 – Allele distance 5

This revealed, that the increase in L3 strains was primarily driven by patients originating from Eastern Africa, particularly Eritrea (Additional file 1: Table S3), which indicates that changes in lineage distribution over time reflected demographic changes rather than successful establishment of strains of newly introduced lineages.

To investigate this further, we performed cluster analyses using a d5 cgMLST threshold as a proxy for recent transmission [25, 26]. Overall, 1,062 of 3,131 strains (34%) were grouped into 269 genomic clusters (average cluster size 3.9, Table 2).

Although strains from all major lineages were detected in the study population, clustering was strongly associated with L4 strains (chi-square test, *p* < 0.001). Of the 269 identified clusters, 218 (81%) were formed by L4 strains, whereas only 22, 21, 6 and 2 clusters were attributed to L3, L2, L1 and L6 strains, respectively. Likewise, the proportion of clustered strains was highest for L4 (39%) compared with L3 (22%), L2 (26%) and L1 (8%; Table 2).

**Fig. 3:**
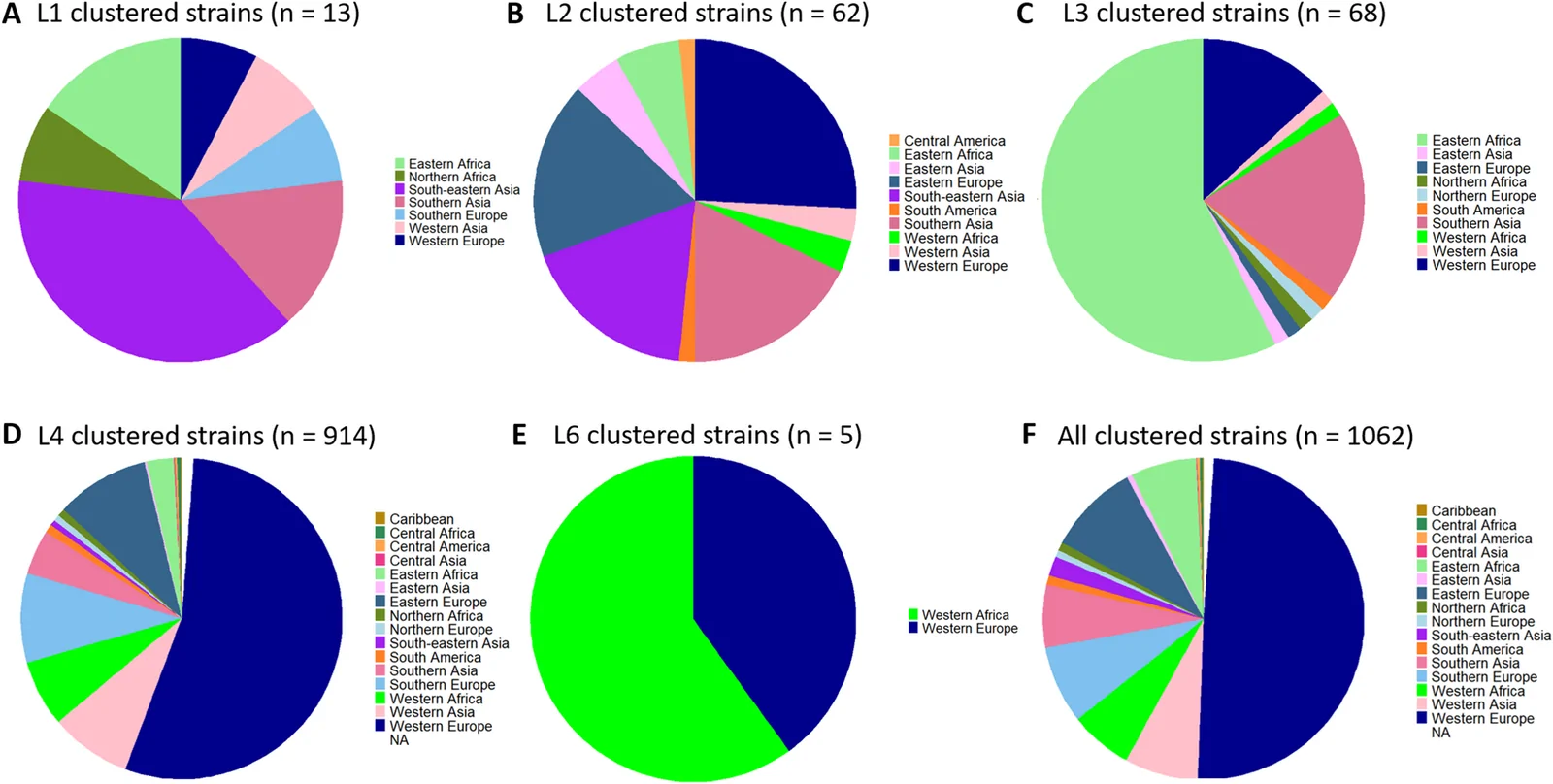
Variability of the self-reported ancestry of patients that have been infected by a clustered strain per *Mycobacterium tuberculosis* complex (Mtbc) lineage in Hamburg, Germany, 1997–2021, *n* = 1,062. Regions are defined according to United Nations statistics division and displayed in the colour indicated in the legend

To investigate whether transmission patterns differed according to the patients’ geographical origin, we analysed the self-reported ancestry of patients infected with clustered strains, classified according to United Nations-defined geographic subregions (Fig. 3, Additional file 1: Table S4). Most clustered L4 strains were isolated from patients with European ancestry (54%, *n* = 498), whereas clustered L3 strains were predominantly associated with patients originating from Eastern Africa (58%, *n* = 39) and Southern Asia (19%, *n* = 13). The strains of the largest L3 cluster (*n* = 19) were mainly isolated from patients born in Eritrea (16/19, 84%, Eastern Africa, Additional file 1: Table S5). Clustered L2 strains were mainly observed among patients with European (44%, *n* = 27) and Asian (44%, *n* = 27) ancestry (Fig. 3). Clustered L1 strains were mostly isolated from patients born in Asia (62%, 8/13, Fig. 3A) and clustered L6 strains from patients born in Western Africa (60%, 3/5, Fig. 3). It is noteworthy that L2 strains were present from the very beginning of the study (Fig. 1A) but did not show increased spread or clustering, even though they had a broader host range comparable to that of the L4 strains, and previous studies had suggested increased virulence [24, 32, 33].

To place the Hamburg strains into a broader global context, we compared representative strains from major clusters (Cluster 1–2 and Cluster7) with publicly available genomes from EnteroBase (Additional file 2: Figure S1). Strains of the major L4 clusters primarily connected to publicly available genomes originating from Germany. In contrast, the strains from the largest L3 cluster, which was associated with patients born in Eritrea in our study, showed genetically related genomes from several countries represented in EnteroBase (Additional file 2: Figure S1).

These findings suggest that, while migration continuously introduced genetically diverse Mtbc strains into Hamburg, local transmission remained largely driven by established L4 strains circulating within the resident population.

**Fig. 4:**
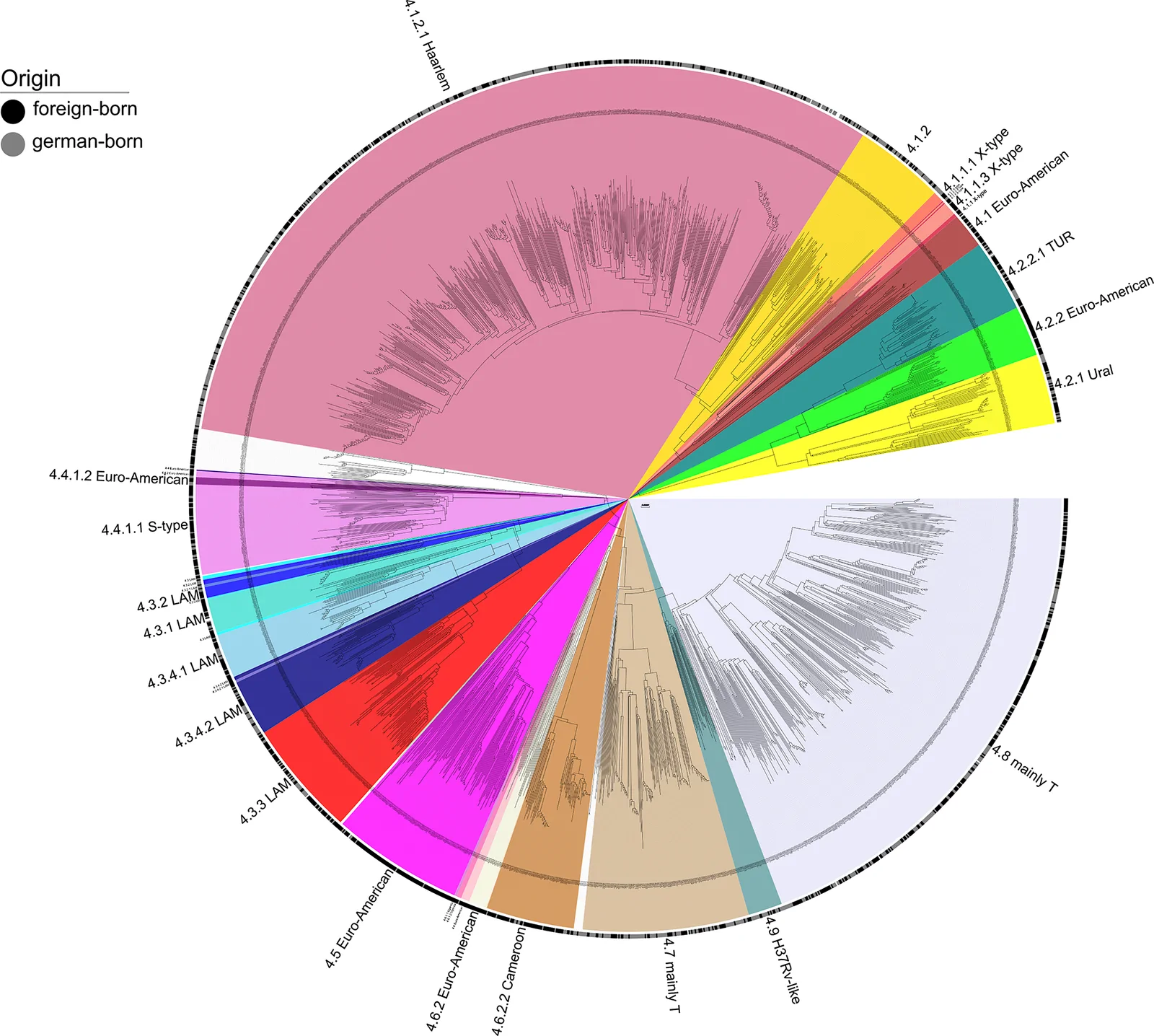
Maximum-likelihood tree of *Mycobacterium tuberculosis* complex Lineage 4 sublineages in Hamburg, Germany, 1997–2021. A maximum likelihood phylogenetic tree (substitution model K3Pu + F+ASC+R2 as automatically determined by ModelFinder Plus of IQ-TREE 2, 1,000 bootstrap replicates) was calculated from the concatenated SNP alignment of 24,658 informative sites. Branches are color-coded according to sublineage and labelled at the edge. The outer ring shows origin: grey – German-born; black – foreign-born

To understand why L4 strains dominated transmission despite increasing importation of strains of diverse Mtbc lineages, we performed an in-depth analysis of L4 sublineages. We classified L4 strains into 21 sublineages based on the Coll et al. [10] classification. The most common sublineages were strains of L4.1.2.1 (32%, 743/2,337), L4.8 (20%, 476/2,337), and L4.3 (11%, 248/2,336; Fig. 4, Additional file 1: Table S6).

Strains of several sublineages, including L4.1.2.1 and L4.7, were primarily found in German-born patients, while strains of other sublineages such as L4.2.2, L4.5, and L4.6.2.2 were predominantly associated with patients born abroad (Fig. 4, Additional file 1: Table S6).

Strains of sublineages L4.2.2.1, L4.7, L4.6.2.2 and L4.8 showed high proportions of clustered strains (57%, 50%, 48%, and 41% respectively, Additional file 1: Table S6), while strain of others such as L4.5 (17%) or L4.4, L4.4.2, L4.6.1, and L4.6.2 (0%) showed little or no clustering (Additional file 1: Table S6). While most L4 clusters were small (≤ 3, 149/218, 68%), five large clusters (> 20 strains) were identified (Fig. 3). Those clusters were formed by strains of L4.1.2.1 (four clusters) and L4.8 (one cluster), and included two clusters that persisted throughout the 25-year study period, underlining the epidemiological success of these strain types (Additional file 1: Table S5). Despite the long-term persistence of strains of these major clusters, the composition of the L4 population remained dynamic: the proportion of L4.8 strains for example increased significantly over time (Mann-Kendall Trend Test, *p* = 0.004, z = 2.92), while proportions of L4.1.2.1 strains declined (*p* = 0.01, z = -2.52, Fig. 1D). In some years, such as 2013 and 2021, L4.8 strains surpassed L4.1.2.1 strains in frequency.

Interestingly, strains of particular sublineages such as L4.1.2.1, L4.8, and L4.3 were detected in patients born in over 15 regions each, with over 57% of cases from Europe – supporting their representation as generalist lineages with global success (Additional file 2: Figure S2, Additional file 1: Table S7). Conversely, strains of other sublineages such as L4.5 and L4.2.2 showed more geographically restricted distributions. For instance, 79% of L4.5 strains were found in patients born in Southern Asia, and L4.2.2.1 was most common among individuals born in Western Asia (46%) or Western Europe (38%). Although most patients infected with a L4.6.2.2 strain were from Western Africa (51%), others were from Western Europe (20%), Central Africa (8%), and 11 other regions, suggesting a broader host range than previously assumed [17].

Consequently, imported strains contributed substantially to population diversity but only a subset contributed to local transmission.

## Discussion

This 25-year longitudinal study examined the population structure and transmission dynamics of Mtbc strains in Hamburg, using molecular data and self-reported ancestry of patients. This study provides unique insights into Mtbc strain population dynamics in a low-incidence metropolitan setting and allows us to further study the potential “spillover risk” by introducing Mtbc strains from high-incidence settings via migration. Although migration substantially altered the composition of the Mtbc population structure in Hamburg over the 25-year study period, these changes primarily reflected repeated introductions of strains of particular lineages rather than sustained local transmission. Instead, transmission remained dominated by well-established L4 strains, demonstrating that changes in population structure do not necessarily translate into changes in transmission dynamics. Specifically, L2 strains, for which a high transmission potential has been observed in other areas of the world [24, 32, 33], remained a minor infecting strain type over time in our study population and did not form larger clusters among German-born patients (Fig. 1A; Table 2). Notably, no relevant spread to the German-born population in Hamburg has occurred.

Still, our data confirm the generalist/specialist lineage concept as Mtbc strains of some L4 lineages could be found in people from several geographical regions, while strains from more specialized lineages are restricted to people with a confined geographical background. Importantly, our data clearly show, that this concept must be considered at the sublineage level, especially for L4, as strains of some sublineages show high cluster rates and global occurrence, while others do not.

Based on these data, we hypothesise, that the global success of L4 may be driven by only a subset of its sublineages. Strains belonging to sublineages L4.1.2.1, L4.3, and the still poorly characterized L4.8 showed a particularly broad geographic distribution, whereas strains of other L4 sublineages were predominantly isolated from patients originating from a limited number of geographical settings (Additional file 2: Figure S1). Strains of these widespread sublineages formed large clusters and were continuously detected in patients from diverse geographical backgrounds (Additional file 1: Tables S5 and S6). Interestingly, we observed similar proportions of L4.8 and L4.1.2.1 strain infections, with the prevalence of L4.8 increasing over time (Fig. 1D, Additional file 1: Table S6). In contrast to L4.1.2.1 strains, which likely originated in Central Europe [34], strains of several other sublineages evolved elsewhere [35] and may therefore remain better adapted to their local host populations. These findings identify L4.8 strains as a potentially important contributor to both the global dissemination of L4 and local TB transmission in Hamburg, warranting further investigation of its pathobiology. Comparative studies of virulence, adaptation, and host–pathogen interactions across L4 sublineages are urgently needed to better understand the biological determinants of transmission success.

Our findings are supported by a recent population-based genomic study from Taiwan, which demonstrated marked heterogeneity in TB transmission and spatial clustering among circulating Mtbc strains [36]. Despite substantial genetic diversity, only a subset of strain types disproportionately contributed to recent transmission, with L2 strains exhibiting the highest clustering rate. Together with our observations in Hamburg, where migration continuously introduced genetically diverse strains, yet sustained transmission remained largely confined to strains of specific L4 sublineages, these findings suggest that transmission success is determined not only by strain introduction, but also by the interaction between Mtbc phylogeny, host population characteristics, and the local epidemiological setting. Our data further indicate that these differences should be considered at the sublineage level, particularly within L4, where strains closely related sublineages differed markedly in their transmission success and global occurrence.

Comparison with publicly available global genomes revealed distinct patterns among strains from different lineages. Whereas the major Hamburg L4 clusters were predominantly linked to genomes from Germany, consistent with sustained regional transmission, the large Eritrea-associated L3 cluster showed close genetic relationships to strains from multiple countries represented in EnteroBase. This pattern is consistent with migration-associated dissemination of successful L3 strains across Europe and beyond. However, the limited availability of representative genomic data from Eritrea currently precludes more detailed phylogeographic inferences.

This study has several limitations. First, we used country of birth as a proxy for host ancestry, which may not accurately reflect genetic ancestry, particularly in populations with a high proportion of second-generation immigrants. However, we expect this limitation to have only a minor impact given the large cohort size. Second, transmission events occurring outside Hamburg may have been missed. Third, although the five-allele cgMLST threshold is a well-validated proxy for recent transmission, genomic clustering alone cannot establish direct epidemiological links between patients [25]. Finally, host factors influencing susceptibility to infection and transmission remain poorly understood. Nevertheless, our findings, together with previous work [37], support the concept that long-term host–pathogen co-evolution has resulted in lineage-specific differences in transmissibility, with transmission success determined by the interaction between Mtbc lineage and host population, providing a biological basis for the generalist–specialist hypothesis.

## Conclusions

Overall, our findings indicate that migration substantially increases the genetic diversity of Mtbc populations in low-incidence settings, but does not necessarily lead to sustained local transmission of newly or repeatedly introduced strains. Instead, transmission remains largely driven by well-established locally circulating strain types. L4 strains, particularly sublineages L4.1.2.1 and L4.8, dominate locally and show potential for global spread, highlighting their generalist characteristic and a wider host adaptation. Our findings support the generalist/specialist concept at the sublineage level and emphasize the need for further research into host-pathogen interactions to inform targeted, (sub)lineage-specific TB control strategies.

These findings highlight the importance of integrating genomic surveillance with demographic information to distinguish strain importation from ongoing local transmission and to guide TB control in increasingly mobile populations.

## Supplementary Information

Below is the link to the electronic supplementary material.

Supplementary Material 1: Supplementary Tables S1 to S7: Table S1: Counts and proportions of strains of major lineages per 5-year steps. Table S2: Count of patients per region of birth of all strains included. Table S3: Counts of patients infected with a L3 strain per year. A: Patients allocated to their region of birth; B: Patients allocated to their counties of birth. Table S4: Counts of patients per lineage and region of birth. A: all strains; B: clustered strains. Table S5: Metadata table with analysis results. Table S6: Description of the study population infected with a L4 strains and L4 population structure, subdivided for L4 sublineages. Table S7: Regions of birth of patients infected with a L4 sublineage strains; A: all strains; B: clustered strains

Supplementary Material 2: Supplementary Figures S1 and S2: Figure S1. Comparison of representative strains of major clusters with publicly available genomes from EnteroBase. Figure S2. Sublineage distribution of Lineage 4 Mycobacterium tuberculosis complex (Mtbc) strains at the patients’ self-reported ancestry grouped according to United Nations statistics division (https://unstats.un.org/unsd/methodology/m49).

## Data Availability

All data is stored at the European Nucleotide Archive (ENA) under accession number: PRJEB9680 [38]. Experiment Accession numbers per sample are listed in Additional file 1: Table S5.

## Abbreviations

cgMLST: Core genome multilocus sequence typing
CTAB: Cetyltrimethylammonium bromide
d5: Five–allele distance threshold
IQR: Interquartile range
L: Lineage
L1 to L6: Lineages 1 to 6 of the Mycobacterium tuberculosis complex
L4.x: Sublineages of Lineage 4
ML: Maximum likelihood
Mtb: Mycobacterium tuberculosis
Mtbc: Mycobacterium tuberculosis complex
ST: Sequence type
SNP: Single nucleotide polymorphism
TB: Tuberculosis
WGS: Whole genome sequencing
