# Supplementary material for: Migration and tuberculosis transmission in Hamburg, Germany: insights from 25 years of molecular epidemiology
Source: Genome Med. 2026 Sep 17;18:137. doi: 10.1186/s13073-026-01750-7 (PMC13595784; doi:10.1186/s13073-026-01750-7)
Supplement: Supplementary file 1 [file 13073_2026_1750_MOESM2_ESM.docx]

**Figure S1**. Comparison of representative strains of major clusters with publicly available genomes from EnteroBase. **A**. Minimum spanning tree of Cluster 1 strains and related genomes available in EnteroBase. Color coded by “country of origin”; darkblue – Germany, lightblue – United Kingdom. **B**. Minimum spanning tree of Cluster 2 strains and related genomes available in EnteroBase. Color coded by “country of origin”; darkblue – Germany. **C**. Minimum spanning tree of Cluster 7 strains and related genomes available in EnteroBase. Color coded by “country of origin”; darkblue – Germany, lightblue – United Kingdom, orange – Italy, white - missing.
 **Figure S2.** Sublineage distribution of Lineage 4 Mycobacterium tuberculosis complex (Mtbc) strains at the patients’ self-reported ancestry grouped according to United Nations statistics division (<https://unstats.un.org/unsd/methodology/m49>). Each pie chart represents the population structure of Mtbc strains isolated from patients that had their self-reported ancestry in the respective region. Case numbers of each region are indicated below each pie chart.
